# Research priorities for harnessing plant microbiomes in sustainable agriculture

**DOI:** 10.1371/journal.pbio.2001793

**Published:** 2017-03-28

**Authors:** Posy E. Busby, Chinmay Soman, Maggie R. Wagner, Maren L. Friesen, James Kremer, Alison Bennett, Mustafa Morsy, Jonathan A. Eisen, Jan E. Leach, Jeffery L. Dangl

**Affiliations:** 1 Department of Botany and Plant Pathology, Oregon State University, Corvallis, Oregon, United States of America; 2 Department of Natural Resources and Environmental Sciences, University of Illinois at Urbana Champaign, Urbana, Illinois, United States of America; 3 Department of Plant Pathology, North Carolina State University, Raleigh, North Carolina, United States of America; 4 Department of Plant Biology, Michigan State University, East Lansing, Michigan, United States of America; 5 Program in Ecology, Evolutionary Biology and Behavior, Michigan State University, East Lansing, Michigan, United States of America; 6 MSU-DOE Plant Research Laboratory, Michigan State University, East Lansing, Michigan, United States of America; 7 The James Hutton Institute, Invergowrie, Dundee, Scotland; 8 College of Natural Sciences and Mathematics, University of West Alabama, Livingston, Alabama, United States of America; 9 Genome Center, University of California, Davis, California, United States of America; 10 Bioagricultural Sciences and Pest Management, Colorado State University, Ft Collins, Colorado, United States of America; 11 Howard Hughes Medical Institute, Department of Biology, University of North Carolina at Chapel Hill, Chapel Hill, North Carolina, United States of America

## Abstract

Feeding a growing world population amidst climate change requires optimizing the reliability, resource use, and environmental impacts of food production. One way to assist in achieving these goals is to integrate beneficial plant microbiomes—i.e., those enhancing plant growth, nutrient use efficiency, abiotic stress tolerance, and disease resistance—into agricultural production. This integration will require a large-scale effort among academic researchers, industry researchers, and farmers to understand and manage plant-microbiome interactions in the context of modern agricultural systems. Here, we identify priorities for research in this area: (1) develop model host–microbiome systems for crop plants and non-crop plants with associated microbial culture collections and reference genomes, (2) define core microbiomes and metagenomes in these model systems, (3) elucidate the rules of synthetic, functionally programmable microbiome assembly, (4) determine functional mechanisms of plant-microbiome interactions, and (5) characterize and refine plant genotype-by-environment-by-microbiome-by-management interactions. Meeting these goals should accelerate our ability to design and implement effective agricultural microbiome manipulations and management strategies, which, in turn, will pay dividends for both the consumers and producers of the world food supply.

## Introduction

A growing appreciation of microbial diversity and function in combination with advances in omics (i.e., the study of large-scale biological datasets, including genes, transcripts, proteins and metabolites) and data analytics technologies are fueling rapid advances in microbiome research. One driving motivation—harnessing beneficial microbes and reducing impacts of detrimental microbes—is common to both humans and crop plants. The National Institutes of Health-funded Human Microbiome Project [1] and the parallel European Union effort [2] helped develop resources and define research goals for long-term directed growth in the field. A coordinated research effort for plant microbiomes, analogous to the Human Microbiome Project, is needed to accelerate the integration of beneficial plant microbiomes into modern agricultural practices that are under strain from human population growth and a changing global climate.

Studies to date have explored plant microbiome structure and function under natural and agricultural environments in both model and crop plant species, including *Arabidopsis thaliana* [3–6], barley (*Hordeum vulgare*) [7], soybean (*Glycine max*) [6,8,9], corn (*Zea mays*) [10], wheat (*Triticum aestivum*) [11,12], rice (*Oryza sativa*) [13–15], and cottonwood trees (*Populus trichocarpa*) [16]; however, there has been no coordinated effort to consolidate and translate new ideas into practical solutions for farmers. This stems both from a lack of coordination among academic and industry researchers and from a disconnect between researchers and farmers. Here, we outline a core set of research priorities (Box 1) and discuss how they will contribute to microbiome management strategies designed to enhance the sustainability of food production.

**Box 1. Agricultural microbiome research priorities**

1. Develop model host–microbiome systems for crop plants and non-crop plants with associated microbial culture collections and reference genomes
2. Define core microbiomes and metagenomes in model host–microbiome systems
3. Elucidate the rules of synthetic, functionally programmable microbiome assembly
4. Determine functional mechanisms of plant–microbiome interactions
5. Characterize and refine plant genotype-by-environment-by-microbiome-by-management interactions

Ever since their origin hundreds of millions of years ago, plants have lived in association with microbes. Among the multitude of host functions that microbes influence are nutrient uptake [17–19], defense [20–23], and phenology [24]. Moreover, the metagenomic potential of the plant-associated microbiome could conceivably dwarf the genomic abilities of plants and, thus, represents a vast, largely untapped reservoir for improved host function. For these reasons, integrating beneficial microbiomes into agricultural systems offers the potential to greatly improve the efficiency of crop plant production [25–28].

While beneficial microbial communities in modern agriculture have been underutilized, efforts to integrate individual microbes into agriculture date back to the 1800s, when the U.S. Department of Agriculture recommended inoculations for legume crops, following experiments demonstrating that rhizobium bacteria colonize nodules and fix nitrogen for their plant hosts [29]. In the past few decades, individual microbes have been inoculated onto crop plants to promote growth, nitrogen and phosphorus uptake [30], and disease resistance [31]. However, these efforts have focused almost exclusively on individual microbial strains [32] and have met with variable success that is typically attributed to the complexity of microbial communities and their interactions with the environment in field settings. Here, we argue that the focus should be on understanding and managing the range from single strains, through synthetic consortia, to whole microbiomes for plant benefit.

A crucial step in coordinating the study of whole plant microbiomes in an agricultural context is the development and implementation of protocol standards. The American Academy of Microbiology [33], Unified Microbiome Initiative [34], Report on the Fast-Track Action Committee on Mapping the Microbiome [35], American Phytopathological Society [36], Earth Microbiome Project [37], Genomic Standards Consortium [38], and National Microbiome Initiative [39] have each called for the standardization of data collection, processing, and analysis. This effort is underway with guidance from the U.S. National Institute of Standards and Technology. Many of the same initiatives listed above have also defined knowledge gaps and research priorities in plant microbiome research. The earlier efforts called for the discovery and description of taxonomic diversity, while more recent emphasis has been placed on elucidating the functional roles of microbiomes. The goal of this paper is not to review these efforts; rather, our objective is to communicate a set of research priorities that should specifically accelerate the integration of plant-associated microbiomes into sustainable agriculture. These priorities reflect both basic and applied goals that can be achieved over the next 5–10 years.

In addition to outlining research priorities, we strongly urge that new research in agricultural microbiomes involve farmers at the onset. Farmers are local experts on crops and land, and are, thus, valuable research partners. For example, farmers played an important role in the discovery of disease-suppressive soils that later became the subject of intensive study and interest to managers [21,40]. In addition to incorporating local knowledge into research, establishing open lines of communication with famers will lead to a greater appreciation of farmers’ objectives and, ultimately, to the translation of research to the field.

## Research priorities

### 1. Model plant-microbiome systems

A model organism is a platform for discovery and hypothesis generation. Systems-level interactions among two or more model organisms, such as host-pathogen or host-symbiont relationships, can likewise shed light on broadly applicable mechanisms of disease and/or symbiosis. In addition to tractability, the power of model systems resides in community-accessible resources, including genome annotation projects, curated mutant collections, standardized protocols, central data repositories, and large-scale field experiments. Establishing these resources will enable research on agricultural microbiomes spanning the full spectrum from the petri dish to the greenhouse to the field.

As an emerging community, plant microbiome researchers must both develop new model systems and build upon established systems to incorporate communities of microorganisms. Large-scale microbial isolation efforts and genome sequencing projects will be necessary to establish culture collections, and coordinated community efforts are necessary to develop a set of standardized protocols and growth platforms to maximize the interoperability of experimental data. One example of a successful, yet non-agricultural, model for plant microbiome research is a small flowering angiosperm in the mustard family, *A*. *thaliana*. However, the *Arabidopsis* system has limitations, such as the lack of symbiotic relationships with nodulating nitrogen-fixers and mycorrhizal fungi, as well as genomic and phenotypic differences from important crops, many of which are monocots. Thus, numerous model systems are needed.

A suite of model plant species is required to understand which processes will translate to crops at varying evolutionary distances [41]. Progress has been made towards the establishment of model host–microbiome systems for the legume *Medicago* [42], *Populus* [43], rice [13,15,44–46], *Sorghum* [47], *Miscanthus* [48], maize [49,50], and tomato [51]. All of these model organisms have fully sequenced genomes and growing communities of researchers to extend their utility for microbiome research. Coordinated efforts to establish public resources, such as repositories and curated databases for sequenced culture collections of associated microbiota, are necessary for full maturation of model systems. Collectively, a multidisciplinary community comprising academic and commercial plant microbiome scientists, with support from funding agencies, is necessary to establish effective model systems to elucidate plant-microbiome interactions with maximum interoperability—a critical step towards establishing a knowledge base for sustainable high-yielding agriculture innovation.

### 2. Defining the core plant microbiome and core plant metagenome

In addition to model systems and culture collections, targeted studies of agricultural microbiomes in the field provide key foundational knowledge that can lead to innovation in several ways. First, identification of the “core” microbiome—the set of microbial taxa that are found in most samples of a particular set of plants [3,4,52,53]—will help to identify plant-associated microbes that should be prioritized for further research, inclusion in culture collections, and manipulative experiments. Plant microbiota are highly diverse, yet not all of these microbes play functionally important roles in their host’s biology. Defining the core microbiome enables researchers to filter out transient associations and refine focus on stable taxa with a greater likelihood of influencing host phenotype. Culture-independent surveys (such as sequencing ITS and *16S rRNA* amplicons) of large numbers of microbiomes of the same plant species from a variety of environments—as opposed to very deep sequencing of a few plant microbiomes—would improve progress towards this goal and could be followed up by selective culturing of candidate core microbiota.

Second, in addition to a taxonomically defined core microbiome based on phylogenetic differences, identifying a functional core is important because constituent microbes are likely to be adaptive. The functional core can be identified by using metagenomic and metatranscriptomic approaches to discover shared predicted functions that are likely important for the given set of plants studied [54]. Extrapolation from phylogenetic marker genes such as ITS provides only limited functional information. In contrast, targeted metagenomics of functional genes and shotgun metagenomics give us a better insight into community functional potential [55], while metatranscriptomics, metaproteomics, and metabolomics reveal the functional community phenotype. By combining multi-omic approaches among large sample sizes of plants, we can identify core community functionality and identify the extent of taxonomic functional redundancy.

**Fig 1:**
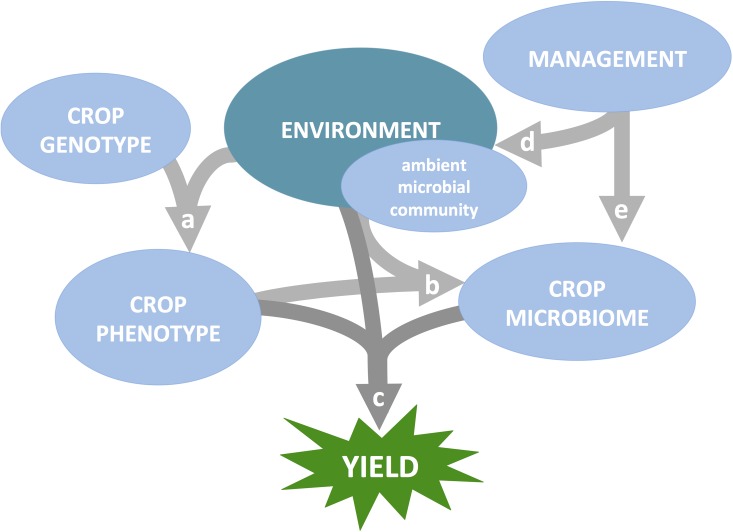
Genotype, environment, management method, and microbiome interact to influence yield. (A) Plant genotype and environmental properties (both biotic and abiotic) synergistically determine plant phenotypes. (B) Plant phenotypic traits influence the subset of microbes from the ambient community—which itself may partly reflect deterministic evolutionary processes like local adaptation to abiotic stresses—that colonize organs to form the crop microbiome. (C) The definition of a “healthy” or “beneficial” microbiome—one that improves yield—likely depends on the particular environmental challenges (both biotic and abiotic) experienced by a plant and the degree to which the plant’s phenotype is already adapted to those challenges. (D, E) Management methods primarily influence yield by altering the dynamics of genotype x environment x microbiome interactions, but can also modify the crop microbiome directly (e.g., microbial seed coating or other microbiome applications that may result from the stated research priorities).

**Fig 2:**
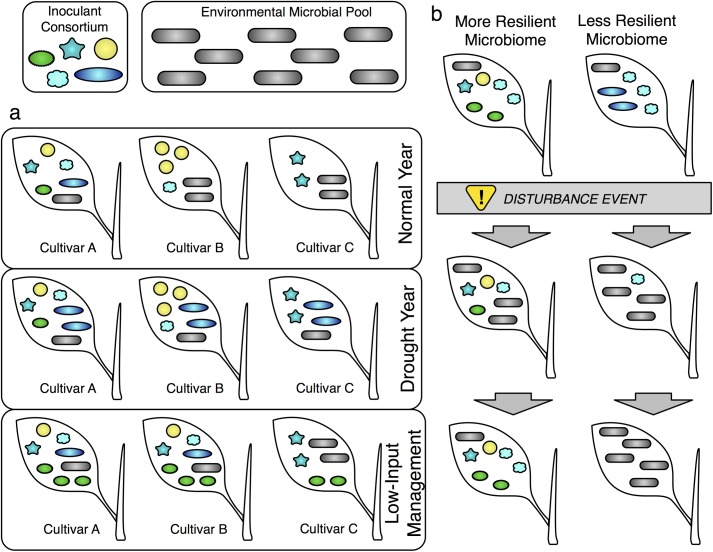
Outcomes of inoculation with a microbial consortium. (A) Illustration of varying outcomes of inoculating with a five-member consortium (colored non-rod shapes) in the presence of a diverse environmental microbial pool (gray rod shapes). Three cultivars are depicted growing in three different regimes: a normal year, a drought year, and low-input management. Genotype effects: In a normal year, Cultivar A is colonized by all five members of the inoculant consortium, while cultivar B is colonized only by yellow spheres and cultivar C is colonized only by teal stars. Environment/management effects: Compared to the normal year, the drought year shows higher colonization by blue ovals and the environmental microbial pool, while under low-input management all cultivars show increased colonization by the green spiky ovals, e.g., a nitrogen-fixer. A genotype-by-environment interaction is depicted by cultivar B only associating with the cyan clouds under low-input management, while cultivar C does not. We note that while interactions between microbes are not shown explicitly, these could be useful in efforts to manipulate microbiomes; e.g., the yellow circles and the cyan clouds always occur together. (B) Temporal dynamics of two communities after a disturbance event (e.g., pathogen attack, high temperature). The more resilient community recovers to its initial state after perturbation, while the less resilient community does not and is displaced entirely by members of the environmental microbial pool. We note that if the growth benefits are provided early in growth by the inoculum, an ecologically fragile community may still be able to enhance crop productivity.

Third, comparing the core microbiomes or metagenomes among key groups of plants or among genotypes of the same plant species could reveal host-driven differences in microbiome assembly. Soil is the major source of microbes that comprise the plant microbiome [52], though some cases of seed-borne vertical transmission have been noted [56], and the atmosphere also contributes to above-ground plant microbiota [57]. Plants of different species or genotypes form largely common core microbiota, at taxonomic ranks of family and higher from the same environmental “inoculum” (Fig 1A, Fig 2A) [4,5,13,24,50,58,59]. Two related questions are: to what extent does the host influence microbiome assembly, and is within-species host genetic variation sufficient for breeding improved microbiome associations? Studies typically find broad-sense heritability of only approximately 5%–7% [24,50], so the ability of microbiome-related traits to respond to classic artificial selection may be limited. However, microbiomes could be engineered to increase heritability [26], and targeted breeding approaches may succeed once we have the foundational knowledge of the host molecular basis of microbiome assembly.

Fourth, comparing the core microbiota of genetically distinct plant groups could reveal plant genes and functional traits that influence microbiome assembly. The mechanisms by which hosts winnow the ambient community to form their microbiota are not fully understood, although plant functional traits such as cuticle composition [60], root length and exudates [44,61], and plant defenses (immunity) [62] have been implicated (Fig 1B). Cuticle and root traits should directly influence colonization by a wide range of microbial species, whereas host–microbe interactions influencing individual microbes (e.g., rhizobia, mycorrhizae) may have indirect effects on later-arriving microbes [63]. In addition, crop species or cultivars that reliably assemble different microbiomes could be relying on their microbiota to fill different needs, especially if the plants are adapted to specific environmental challenges [64].

Finally, because the crop microbiome, plant phenotype, and environment interact to affect yield (Fig 1C), comparing the core microbiomes and metagenomes of plants grown in contrasting environments should provide key insights. Microbes that are especially common in challenging environments are more likely to protect yield under stresses such as drought, heat, salinity, heavy metals, disease, and herbivory (Fig 2A) [65]. For instance, the core metatranscriptomes of plants grown in dry soil could differ from those of plants grown in the same soil under well-watered conditions, providing clues about which microbial genes might contribute to drought tolerance [66]. This approach is particularly powerful when combined factorially with a comparison between stress-resistant and susceptible cultivars in challenging versus unchallenging environments. Additionally, metagenome-wide association studies, in which variants in the metagenome (such as the presence/absence of a certain microbial gene) are used to predict host phenotype [67], could nominate subsets of the microbiome that play key roles in host performance under the major stressors facing agriculture today and in the near future.

### 3. Rules of microbiome assembly and resilience

Beyond knowledge of the identity and functional attributes of microbes present in crop plant “core” microbiomes, determining the rules by which microbes assemble into those communities will be essential for any attempt to manipulate or manage the agricultural microbiome. The extraordinary complexity of plant microbial communities presents a daunting barrier to fully understanding the ecology of plant-associated microbiomes, and for this reason the links between microbiome assembly and plant performance are still poorly understood [68]. Therefore, we recommend prioritizing research aimed at designing synthetic microbial communities that can successfully colonize plant organs and persist long enough in natural environments to confer benefits to the host.

One key question is: what properties of a synthetic microbial community make it more likely to colonize plant organs? Microbial genes that are reliably represented in core metagenomes—and are enriched in plant microbiomes compared to soil—are good candidates for essential functions that improve colonization ability. Community properties such as phylogenetic diversity or species richness might also impact colonization success; these could be systematically explored in the lab using gnotobiotic model systems and culture collections [69]. Once a synthetic community has colonized a plant, what properties allow it to resist invasion and displacement by microbes that are abundant in the surrounding environment? What properties confer resistance to abiotic challenges, or at least the ability to rebound from such perturbations (Fig 2B)? These could be the same as, or different from, the properties that enabled the initial colonization. All of these questions could be initially addressed in gnotobiotic systems in the lab and then tested in greenhouse and field experiments.

In addition to genetic properties of the microbiome, the method of microbiome inoculation can play a major role in its success, as demonstrated by years of agronomic work optimizing delivery of single strains via seed coatings, clay particles, and peat. Development of effective microbiome inoculation strategies for agriculture may include seeding multiple characterized microbial species into resilient particles for delivery via air, water, soil, or novel delivery systems such as insect vectors. However, efforts to maximize colonization ability should also ensure that synthetic communities are not so aggressive that they invade local ecosystems and negatively affect soil health, neighboring plants, or future crops [28].

Finally, what external factors affect the success of a beneficial synthetic microbiome? Synthetic communities may need to be robust to variation in host phenotypic traits that influence assembly, or they may need to be tailored to colonize particular crop species (Fig 1B). Similarly, the colonization ability or resilience of a given community will depend on the environment. Understanding both abiotic (e.g., temperature, light, acidity, nutrient and water availability) and biotic factors (e.g., competition, predation, parasitism, mutualism within the microbiome) influencing microbiome assembly [70] will be essential for any attempt to manage agricultural microbiomes. Testing the influence of biotic factors will be particularly difficult for microbes that cannot be cultured independently. For example, microbes can be “linked” to other microbes by strong interactions (e.g., mycoparasitism). Network analysis can be particularly useful for identifying linked microbes and “hub” microbes, akin to keystone species, that interact strongly with many other microbes and thereby strongly impact both the structure and function of the community [71]. Comparisons of microbiomes across habitats [72,73], the addition or removal of a single biotic component [71,74], dilution experiments [75], and pioneering work manipulating culturable bacteria [62,71] are beginning to provide insight into how biotic interactions influence microbiome assembly and persistence in plants. Ultimately, testing synthetic communities under a variety of abiotic and biotic conditions will improve our ability to predict how these inocula will perform across diverse and variable farm environments (Fig 1C, Fig 2A).

### 4. Functional mechanisms in agricultural microbiomes

Realization of the anticipated benefits of plant microbiomes in agriculture will require a thorough understanding of the functional and mechanistic aspects of the interactions between microbes, plants, the farm environment, and agricultural management practices. Moreover, enabling the engineering of agricultural plant–microbiome associations for improvements in productivity, tolerance to biotic and abiotic stresses, and nutrient efficiency [28,76] will require significant advances in experimental approaches, advanced characterization techniques, modeling, and theory that incorporate, but also move beyond, the currently dominant census-based descriptive approaches [77,78]. The molecular biology and physiology of the vast majority of microbes remains uncharacterized. Also lacking are the empirical and theoretical bases for predicting microbial community function from structure and the functional responses to environmental variation. New high-throughput techniques that enable rapid multi-omics characterization of the functional responses at multiple scales, from individual strains and operational taxonomic units to local microbial communities, across environmental variation, are essential for addressing these knowledge gaps. Bioinformatic and modeling tools capable of capturing the multi-omics manifestations of the interactions between the elements at various scales are also essential for sufficiently understanding and effectively utilizing these interactions [79,80].

Understanding the functional aspects of interactions between plants and their microbiomes is particularly essential for improving agricultural applicability, such as for tolerance to drought and other abiotic stresses as well as resistance to diseases [18,81,82]. Model experimental systems are essential for elucidating the dynamic feedbacks between plant physiology and microbial functions that drive colonization of the rhizosphere, rhizoplane, and endophytic compartments as well as the maintenance and modulation of plant–microbiome interactions. Additionally, large-scale efforts are needed to characterize the role of exudates in shaping these microbial communities and maintaining their functionality [62,83].

Finally, while functional attributes of a few plant-microbe interactions, both pathogenic and beneficial, have been studied in detail and have yielded stunning breakthroughs in our molecular definition of the plant immune system, this research has focused largely on characterizing one-on-one interactions. Studying these interactions in the context of the structure and function of microbial communities will lead to a more complete understanding of these interactions and could lead to potential mechanisms for controlling disease in a manner facilitated by the principles of microbial ecology [84]. For example, in some cases the order of arrival into a microbial community (i.e., ecological priority effects) determines whether an endophyte inhibits or facilitates a pathogen [85]. Discovering community-level characteristics that precede potential pathogenic or beneficial interactions in constructed synthetic communities as well as in wild soil ecosystems could also lead to diagnostic and predictive tools for farmers and regulatory agencies.

### 5. Plant genotype × microbiome × environment × management interactions

The previously discussed goals of defining core microbiomes, identifying functional mechanisms of beneficial symbioses, and discovering the rules of microbiome assembly share a common theme: sensitivity to host genetic and environmental context, as well as management decisions. For instance, the definition of a “healthy” or “beneficial” microbiome may depend on the specific environmental challenges faced by the plant (Fig 1C). On the other hand, microbiomes with generic growth-promoting properties may increase overall plant vigor and improve the host’s ability to cope with a wider range of challenges. Whether globally beneficial microbiomes can be engineered to provide robust benefits to crop health across diverse environments, or whether in-situ engineering of locally beneficial microbiomes should be our goal, will need to be determined. To some extent, the environment experienced by a plant can be controlled by management decisions tailored to the natural challenges presented by a given farm (Fig 1D). Humans have altered biotic and abiotic properties of soils since the start of agriculture through soil additives, tillage, and cropping systems. Thus, the interactions among microbes, the environment, and management are a crucial consideration when applying microbiome science to improve plant health and yield (Fig 1, Fig 2).

Compounding the challenges of implementing beneficial microbiomes for agriculture are genotype-by-environment interactions. Host genotype effects on the microbiome can vary in strength among environments and even between different plant tissues (Fig 1A) [50,86]. Another challenge is to design microbiome treatments that are resilient to the tremendous environmental variability among farms. A given microbial consortium might thrive in one climate or soil type but fail in a different environment to which it is poorly adapted; for instance, microbial diversity can be driven by simple factors such as soil pH (Fig 1B) [87]. A beneficial microbiome’s resilience may also depend on its competitive ability relative to the surrounding microbial community, which can vary dramatically among farms and in response to management practices [88] and climate change [89,90]. Therefore, an overarching challenge is to define microbial consortia that can persist in a variety of heterogeneous ecosystems.

Well-designed, targeted experiments that can disentangle host genotype **×** environment **×** microbiome **×** management interactions are needed to inform management decisions. For instance, to better understand what microbiome or metagenome properties provide maximum benefit under various contexts (Fig 1C), artificial microbiome selection under host–microbiome co-propagation could be performed by manipulating individual environmental stressors (e.g., drought or pathogen presence) and then studying and propagating the microbiomes associated with the healthiest plants [26]. To better understand context-dependence of microbiome assembly (Fig 1B), systematic manipulation of host genotype (e.g., comparing commonly grown cultivars) and abiotic factors (e.g., ambient temperature and soil nitrate) could reveal patterns that enable testable predictions of on-farm microbiome behavior. The use of several distinct ambient communities in these experiments—e.g., simplified “synthetic” or model communities—would lead to more reproducible and generalizable results. Ideally, these results should be validated in the field, across a range of farms, climates, soil types, and management methods.

Plant genotype **×** environment **×** microbiome **×** management interactions are practical challenges to the successful integration of beneficial microbiomes into breeding and agricultural management programs (Fig 1). However, the problem is not out of the reach of careful, systematic experimentation. The development and application of new molecular methods, model systems, and analytical tools will accelerate progress in this crucial area and enable translation of basic discoveries to farmers’ fields.

## Conclusion

More so than ever before, the tools, technologies, and funding [39] are now in place to tackle fundamental questions in agricultural microbiome research. We have outlined five key research priorities in this area (Box 1). By encouraging the pursuit of these goals, we aim to accelerate the development of agricultural microbiome manipulations and management strategies that will increase the sustainability and productivity of global agriculture.
